# Identifying 3^rd^ larval stages of common strongylid and non-strongylid nematodes (class: *Nematoda*) infecting Egyptian equines based on morphometric analysis

**DOI:** 10.1186/s12917-022-03526-8

**Published:** 2022-12-12

**Authors:** Moaz M. Amer, A. Y. Desouky, Nashwa M. Helmy, Ahmed M. Abdou, Sh. S. Sorour

**Affiliations:** 1 Biotechnology Department, Animal Health Research Institute, Dokki, 12618 Egypt; 2 Parasitology Department, Faculty of Veterinary Medicine, Kafrelsheikh University, Kafr El-Sheikh, 33516 Egypt; 3 Forensic Medicine and Toxicology Department, Faculty of Veterinary Medicine, South Valley University, Qena, 83523 Egypt

**Keywords:** Strongylid nematodes, Non-strongylid nematodes, Equine, 3^rd^ larvae, Egypt

## Abstract

Strongylid and non-strongylid nematodes are one of the most important parasites infecting equines. The traditional method to identify these nematodes is through coproscopy and fecal culture. Because of the scarcity of data published in Egypt discussing the morphometric features of infective 3^rd^ larvae of these nematodes, this study aims to provide a morphometric key for L3 of common strongylid and non-strongylid nematodes infecting Egyptian equines. For this reason, we cultured fecal samples containing GINs eggs and 3^rd^ larval stages were identified based on their morphology (i.e., shape and number of intestinal cells (IC), shape of the esophagus, and shape of the tail sheath) in addition to computing their dimensions (i.e., length of larvae with sheath, length of the esophagus, length of intestinal cells, and body breadth). We identified 3^rd^ larval stages of four strongylid nematodes (*Cyathostomum* *sensu* *lato*, *Strongylus vulgaris*, *Strongylus equinus*, and *Strongylus edentatus*) as well as two non-strongylid nematodes (*Strongyloides westeri*, and *Trichostrongylus axei*). Statistically, our results revealed significant differences in terms of total length, body width, esophagus length, and gut length among 3^rd^ larvae identified in the current study. The combination of both morphological and metric keys will allow the better identification of common strongylid and non-strongylid nematodes infecting equines.

**Supplementary Information:**

The online version contains supplementary material available at 10.1186/s12917-022-03526-8.

## Introduction

In many developing economies, including Egypt, they rely more heavily on working equines including horses, donkeys, and mules in rural areas. Regardless of the fact that these equines play significant role in sustaining the daily lives of people by providing support in industries such as agriculture, construction, tourism, and public transportation. However, the health and welfare of domesticated equines is frequently overlooked [1].

Gastrointestinal nematodes (GINs) are one of the parasite groups that cause a variety of clinical and economical health problems among equines [2]. Infection with GINs commonly produces broad-based clinical signs. For instance; anorexia, reduced feed intake, and diarrhea [3, 4] elicit a diminished weight gain and degradation of traction power. Gastrointestinal nematodes infecting equines include; (A) strongylid nematodes, which make up about 75% of the total parasite population infecting equines [5, 6], and (B) Non-strongylid nematodes which include *Parascaris equorum*, *Habronema* spp., *Draschia megastoma*, *Oxyuris equi*, *Trichostrongylus axei* and *Strongyloides westeri* [5].

Strongylid nematodes (Family: *Strongylidae*) are classified into two sub-families; (A) Large Strongyles or Strongylins (Subfamily: *Strongylinae*) which are characterized by having a globular buccal capsule, and (B) Small Strongyles or Cyathostomes (Subfamily: *Cyathostominae*) which characterized by having a cylindrical buccal capsule [3–5, 7–9].

The key difference between large and small strongyles is the existence of extraintestinal migration in the case of large strongyles where L3 of *Strongylus vulgaris* migrates from GIT toward anterior mesenteric artery, L3 of *Strongylus edentatus* migrates toward liver and peritoneum, and L3 of *S. equinus* migrate toward pancreas and liver [3, 4]. Migratory stages of large strongyles contribute to serious health hazards for equines where larvae of *S. vulgaris* set up fatal thrombi that can block major arteries (i.e., cranial mesenteric artery) causing gangrenous enteritis, intestinal stasis, and colic. Thrombi may also detach and block other arteries (i.e., iliac artery) resulting in lameness. Larvae of *S. equinus* create hemorrhagic tracts in the liver and pancreas, while larvae of *S. edentatus* produce hemorrhagic nodules in the peritoneum’s wall, caecum, and colon [3].

Although L3 of small strongyles don’t migrate outside the alimentary tract, they encyst inside the mucosal lining of the cecum and colon where they continue their development then released into the lumen of the cecum and colon giving rise to mature stages [3, 9, 10]. Massive emergence of larval stages of small strongyles from mucosal cysts into the lumen of cecum and colon especially in warm weather (late winter to early spring) triggers a clinical case called “Cyathostominosis” where infected animal suffers from emaciation, colic, diarrhea, subcutaneous edema, pyrexia, and fatality in uncontrolled cases [5, 9, 10].

*Strongyloides westeri* (Family: *Strongyloididae*) is a common parasite with a direct life cycle where adult worms inhabiting the small intestine of young equines (up to 4 months of age) who get infected either during suckling or through percutaneous invasion that’s followed by pulmonary migration. Pathological lesions due to *S. westeri* infestation like enteritis characterized by erosions, catarrhal lesions, and mucosal edema are only executed by female worms of *S. westeri* that are mirrored clinically by impaired digestion, malabsorption, and diarrhea [5].

*Trichostrongylus axei* (Family: *Trichostrongylidae*) is a parasite inhabiting the fundus area of the equine stomach [11]. Infective larvae after being ingested by equines will develop in the lumen of mucosal crypts or deeply in the gastric mucosa. Light infection is usually less pathogenic while heavy infection frequently produces a hyperplastic reaction in glandular tissue of the fundus with the formation of raised plaques that ranged from a half-centimeter to several centimeters which later will erode in their centers [11, 12].

Diagnosis of GINs normally relies on discussing the health status of the animal in former times, clinical signs, and microscopical examination of fecal samples [3]. Although microscopical examination of fecal samples has the advantage of being cheap and requires neither expensive reagents nor equipment [13], it is hard to differentiate GINs to species level. Therefore, as a rule, the microscopical examination comes along with fecal culture to identify GINs to species level based on morphological features of infective 3^rd^ larval stages [3].

In Egypt, there is a paucity of data discussing morphometric characters of 3^rd^ larval stages of GINs infecting equine. Therefore, the present work aims to identify and differentiate L3 of common strongylid and non-strongylid nematodes to species level based on their morphometric features.

## Material and methods

### Samples

Over 1 year period (from October 2015 to September 2016), we randomly collected 260 fecal samples from donkeys and horses from different spots in Great Cairo (Giza Zoo, Brooke hospital, and Al-Zahra Stud). Samples were collected from the rectum in sterile plastic packs and sent directly to Animal Health Research Institute, Dokki, Egypt. All samples were prepared and examined on the same day of collection.

### Coprological examination

According to previous reported study [14], fecal samples were examined by fecal flotation test using saturated NaCl solution. According to the result of the flotation test, we cultured fecal samples containing GINs eggs in wetted sawdust for 7 days and incubated at 27 °C to allow the development of infective 3^rd^ larvae. Subsequently, we gathered 3^rd^ larvae up on funnels using the Baermann technique, stained them with 2% Lugol’s iodine solution, and identify them under the microscope.

We classified cultured 3^rd^ larvae under the microscope according to previous reported study [15] based on the following morphological parameters; (1) shape of the esophagus, (2) number and shape of gut cells, (3) shape of the tail sheath. Together with morphological identification, we calculated the following dimensions according to [16]; (A) length of larvae with sheath, (B1) length of esophagus, (B2) length of intestinal cells, and (C) body width using a stage micrometer calibrated according to [17].

### Statistical analysis

GraphPad Prism 8.3.4 software (GraphPad Software Inc., La Jolla, CA, USA) was used for data analysis. Data are presented as the mean ± standard deviation (SD). Statistical analyses were performed using one-way analysis of variance (ANOVA) followed by the Tukey-Kramer hoc test for group comparisons. Statistically significant differences, defined by *p*-values of < 0.05, are marked in the figures with asterisks and defined in each corresponding figure legend together with the name of the statistical test that was used.

## Results

**Fig. 1:**
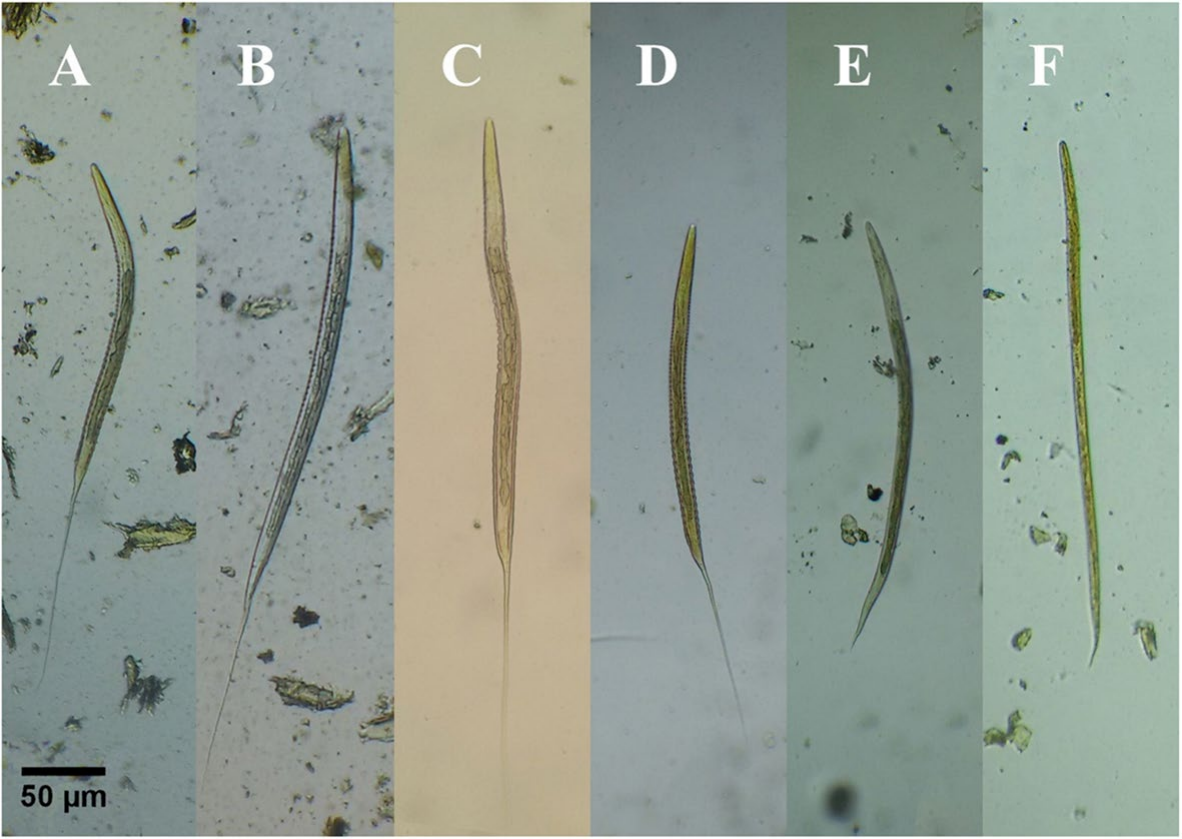
L3 of GINs infecting equine (Stained with 2% Lugol’s iodine, × 10 magnification, bar = 50 μm). **A** *Cyathostomum* *sensu* *lato*. **B** *Strongylus vulgaris*. **C** *Strongylus equinus*. **D** *Strongylus edentatus*. **E** *Trichostrongylus axei*. **F** *Strongyloides westeri.* All images were taken by the authors

Our findings revealed that GIN eggs were detected in 53.1% of the tested samples (138/260). We identified infective third larval stages of four strongylid nematode species (*Cyathostomum* *sensu* *lato*, *Strongylus vulgaris*, *Strongylus equinus*, and *Strongylus edentatus*), as well as two non-strongylid nematode species (*Trichostrongylus axei*, and *Strongyloides westeri*) using coproculture technique (Fig. 1). In the current study, *Cyathostomum s.l.* was the most common larvae, while *T. axei* was the least common (data not shown).

### Total length

**Fig. 2:**
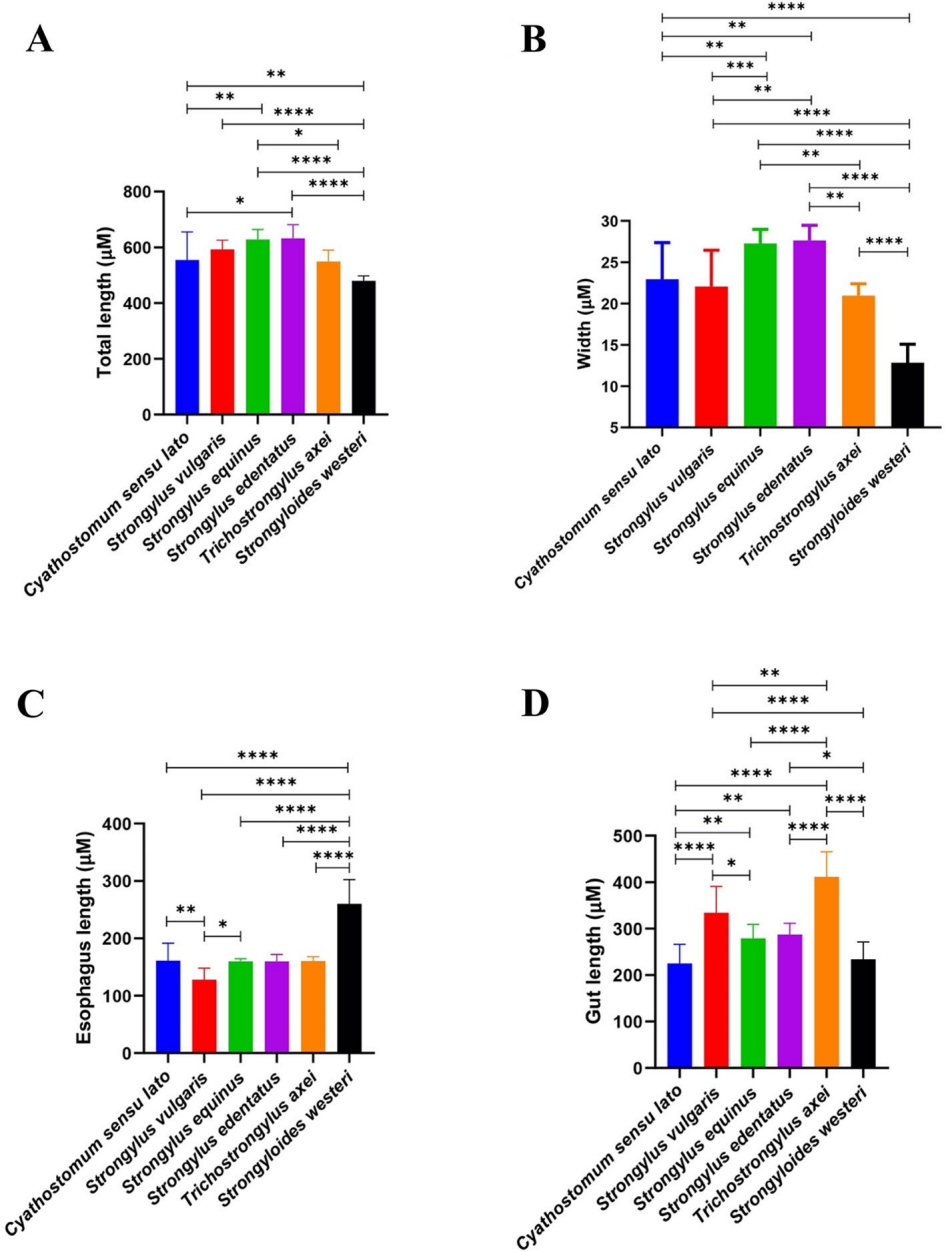
Statistical analysis of different metrics used to identify L3 of equine GINs. Samples were collected over 1 year period from October 2015 to September 2016. Values are presented as the means ± SD. Statistically significant differences were determined by a one-way ANOVA and a Tukey-Kramer post hoc analysis (*P < 0.05). **A** Total length of *Cyathostomum s.l.* (*N* = 18); *S. vulgaris* (*N* = 14); *S. equinus* (*N* = 13); *S. edentatus* (*N* = 9); *T. axei* (*N* = 7); *S. westeri* (*N* = 16). **B** Body width of *Cyathostomum s.l.* (*N* = 19); *S. vulgaris* (*N* = 16); *S. equinus* (*N* = 13); *S. edentatus* (*N* = 9); *T. axei* (*N* = 7); *S. westeri* (*N* = 18). **C** Esophagus length of *Cyathostomum s.l.* (*N* = 19); *S. vulgaris* (*N* = 16); *S. equinus* (*N* = 13); *S. edentatus* (*N* = 9); *T. axei* (*N* = 7); *S. westeri* (*N* = 18). **D** Gut length of *Cyathostomum s.l.* (*N* = 19); *S. vulgaris* (*N* = 16); *S. equinus* (*N* = 13); *S. edentatus* (*N* = 9); *T. axei* (*N* = 7); *S. westeri* (*N* = 17)

Our result show that total length of L3 of both *S. equinus* and *S. edentatus* are significantly the longest (628.1 μm ± 36 and 632.2 μm ± 48.5 respectively), while those of *S. westeri* are significantly the shortest (479.8 μm ± 17.7). Moreover, the mean length of L3 of *S. vulgaris*, *Cyathostomum s.l.*, and *T. axei* show non-significant borderline values (592.6 μm ± 33, 554.6 μm ± 100.5, and 549 μm ± 40.5 respectively). Differentiation of L3 of large strongyles based on total length showed non-significant difference. However, total length of *S. equinus* and *S. edentatus* is significantly higher than *Cyathostomum s.l*. 3^rd^ larvae. Finally, the third larvae of *T. axei* only exhibit a significant difference compared to the L3 of *S. equinus* (Figs. 1 and 2A, Table 1A, S1).

**Table 1:** Morphometric features of 3^rd^ larval stages of strongylid and non-strongylid nematode species identified by fecal culture

	***Cyathostomum s.l.***	***S. vulgaris***	***S. equinus***	***S. edentatus***	***T. axei***	***S. westeri***
**A) Total Length**						
Mode (μm)^A^	370	608	605	562	487	471
Median (μm)	606.5	602.5	628	624	556	474.5
Minimum (μm)	370	521	563	562	487	457
Maximum (μm)	670	650	686	704	594	514
Mean (μm)	554.6	592.6	628.1	632.2	549	479.8
SD	100.5	33	36	48.5	40.5	17.7
SE	23.7	8.8	10	16.2	15.3	4.4
CV	0.2	0.1	0.1	0.1	0.1	0.04
MAD	34	16	23	38	28	11
Skewness	−1	−0.6	0.02	0.3	−0.7	0.8
SES	0.5	0.6	0.6	0.7	0.8	0.6
**B) Body Width**						
Mode (μm)^B^	23	26	26	27	21	14
Median (μm)	24	23	27	28	21	14
Minimum (μm)	14	12	24	24	18	7
Maximum (μm)	28	26	31	30	22	15
Mean (μm)	23	22	27	28	21	13
SD	4.4	4.3	1.9	1.9	1.4	2.4
SE	1	1.1	0.5	0.6	0.5	0.6
CV	0.2	0.2	0.1	0.1	0.1	0.2
MAD	2	2	1	1	1	1
Skewness	−1.1	−1.4	0.1	−0.8	−2	−1.8
SES	0.5	0.6	0.6	0.72	0.8	0.5
**C) Esophagus**						
Shape	Filariform	Filariform	Filariform	Filariform	Filariform	Filariform
Mode (μm)^C^	167	134	162	155	152	249
Median (μm)	168	134.5	161	159	160	264.5
Minimum (μm)	101	84	151	134	151	140
Maximum (μm)	194	145	167	180	170	325
Mean (μm)	161	128	160	160	160.4	259.9
SD	30.4	20	4.3	12.5	7.2	42.2
SE	7	5	1.2	4.2	2.7	10
CV	0.2	0.2	0.03	0.1	0.05	0.2
MAD	13	7.5	2	4	7	17
Skewness	−1.2	−1.6	−0.5	−0.7	0.01	−1.4
SES	0.5	0.6	0.6	0.7	0.8	0.5
**D) Gut**						
IC						
Number	8	32	16	18–20	16	Poorly Defined IC
Shape	Triangular IC	Triangular IC	Triangular IC	Indistinct IC	Indistinct IC	
Gut Length						
Mode (μm)^D^	248	321	264	257	465	150
Median (μm)	248	345.5	280	280	425	240
Minimum (μm)	139	190	239	257	310	150
Maximum (μm)	266	398	352	325	465	276
Mean (μm)	225.1	333.6	279.2	287.2	411.3	234.1
SD	41	56.8	29.4	24.3	53.6	37.2
SE	9.4	14.2	8.2	8.1	20.3	9
CV	0.2	0.2	0.1	0.1	0.1	0.2
MAD	15	22	16	18	36	23
Skewness	−0.9	−1.8	1.1	0.4	−1.1	−1.3
SES	0.5	0.6	0.6	0.7	0.8	0.6

^A,B,C,D^Multiple modes exist (only the first one is reported). *SD* standard deviation, *SE* standard error, *CV* coefficent of variation, *MAD* median absolute deviation, *SES* standard error of skewness, *IC* Intestinal Cells, *Cyathostomum s.l Cyathostomum* *sensu* *lato*, *S. vulgaris Strongylus vulgaris*, *S. equinus Strongylus equinus*, *S. edentatus Strongylus edentatus*, *T. axei Trichostrongylus axei*, *S. westeri Strongyloides westeri*

### Body width

In terms of body width, our findings revealed that 3^rd^ larvae of *S. westeri* characterized by their highly significant needlelike body shape (13 μm ± 2.4) while L3 of *S. equinus* and *S. edentatus*, on the other side, characterized by their significant broadened bodies (27 μm ± 1.9, and 28 μm ± 1.9 respectively). Our results showed that L3 of large strongyles are significantly differ in their body width where L3 of *S. vulgaris* have a lower body width (22 μm ± 4.3) compared to *S. equinus* and *S. edentatus*. On the other hand, L3 of *Cyathostomum s.l.* could be differentiated from L3 of *S. equinus* and *S. edentatus* by a significant reduction in their body width (23 μm ± 4.4). Moreover, the body width of L3 of *S. vulgaris* and *Cyathostomum s.l.* differs in a non-significant way (Figs. 1 and 2B, Table 1B, S2).

### Esophagus

**Fig. 3:**
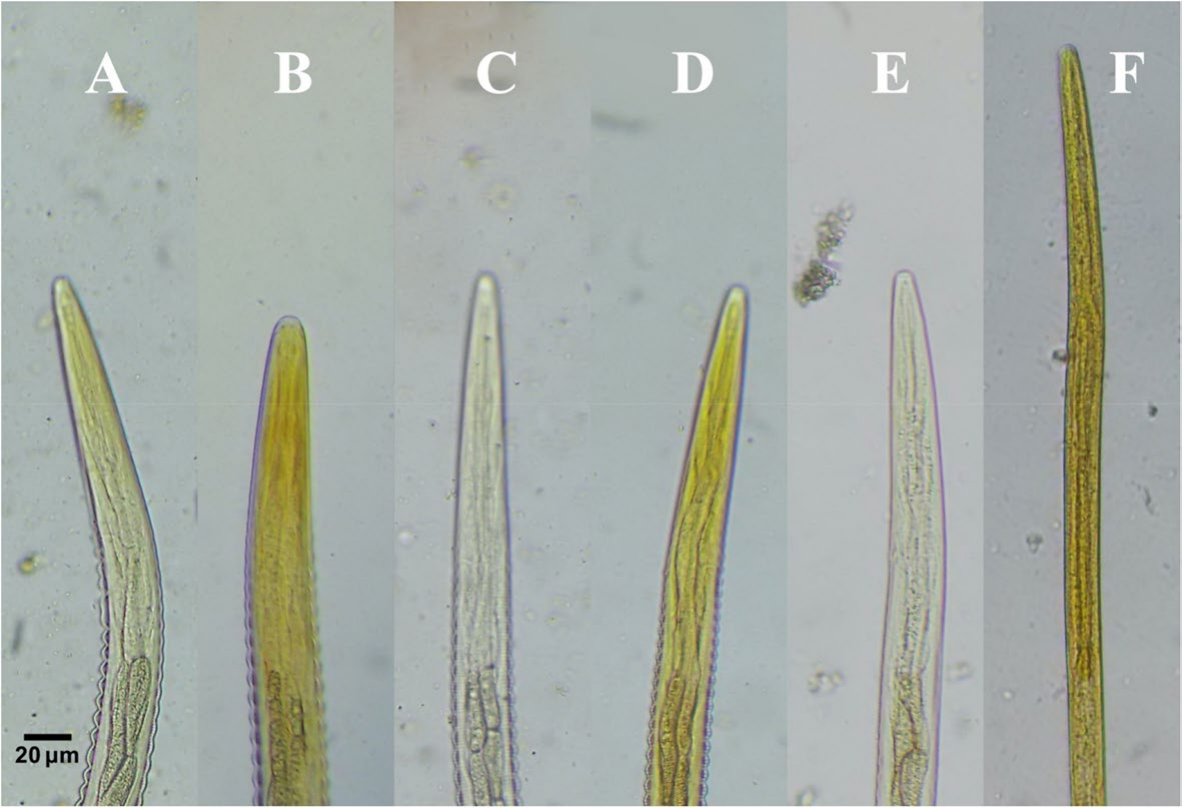
Anterior end showing esophagus of L3 (Stained with 2% Lugol’s iodine, × 40 magnification, bar = 20 μm). **A** *Cyathostomum* *sensu* *lato*. **B** *Strongylus vulgaris*. **C** *Strongylus equinus*. **D** *Strongylus edentatus*. **E** *Trichostrongylus axei*. **F** *Strongyloides westeri.* All images were taken by the authors

Our result showed that L3 of *S. westeri* can be distinguished from other larvae by their significantly elongated shape esophagus (259.9 μm ± 42.2). Moreover, according to our results, the ratio of the esophagus to total body length in case of *S. westeri* were close to 1:2 making these larvae easily to be identified under the microscope based on their unique shaped esophagus (Fig. 3). Furthermore, our results showed that the length of esophagus among L3 of *S. vulgaris* (128 μm ± 20) was significantly lower than L3 of *Cyathostomum s.l*. (161 μm ± 30.4) and L3 of *S. equinus* (160 μm ± 4.3) (Fig. 2C, Table 1C, Table S3).

### Gut

**Fig. 4:**
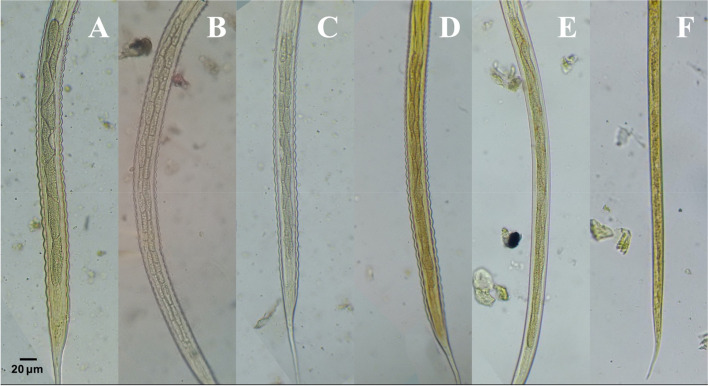
Intestinal Cells of L3 (Stained with 2% Lugol’s iodine, × 40 magnification, bar = 20 μm). **A** *Cyathostomum* *sensu* *lato*. **B** *Strongylus vulgaris*. **C** *Strongylus equinus*. **D** *Strongylus edentatus*. **E** *Trichostrongylus axei*. **F** *Strongyloides westeri.* All images were taken by the authors

The number and shape of intestinal cells (IC) are considered one of the main touchstones we used to differentiate infective 3^rd^ larvae where L3 of *Cyathostomum s.l.* contain 8 triangular IC, *S. vulgaris* contain 32 triangular IC, *S. equinus* contain 16 triangular IC, *S. edentatus* contain 18–20 indistinct IC, *T. axei* contain 16 indistinct IC, while *S. westeri* 3^rd^ larvae have poorly defined IC (Fig. 4).

**Fig. 5:**
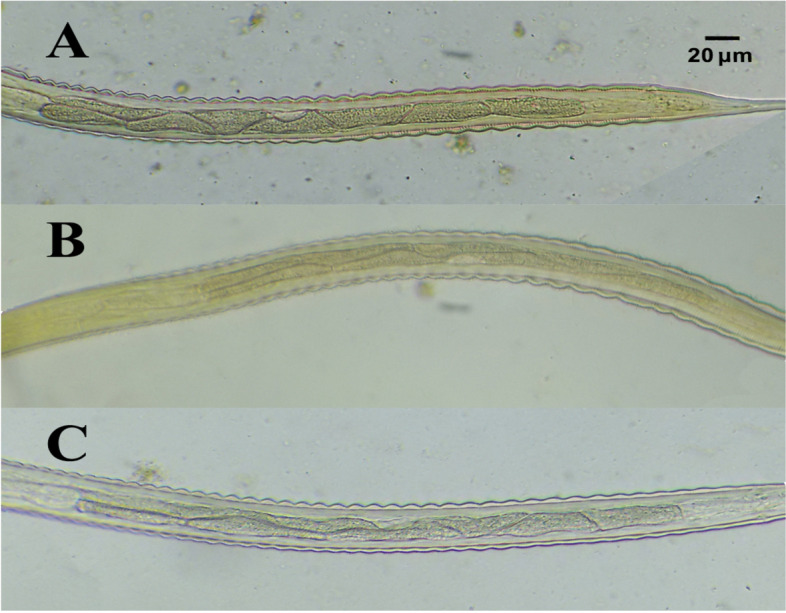
Different L3 of *Cyathostomum* *sensu* *lato* based on the arrangement of Intestinal Cells (IC)*.* ***A*** *Type A Cyathostomum s.l. (*2 + 6 IC). **B** T*ype B Cyathostomum s.l.* (4 + 4 IC). **C** *Type C Cyathostomum s.l.* (2 + 2 + 4 IC). Larvae were stained with 2% Lugol’s iodine, original magnification × 40, bar = 20 μm*.* All images were taken by the authors

We identified three types of *Cyathostomum s.l*. Based on the arrangement of intestinal cells: Type (A) in which the first two cells are arranged in a double row and the remaining 6 cells arranged in a single row, Type (B) in which intestinal cells arranged in double rows each one of them contained 4 cells, and Type (C) in which 1st four intestinal cells arranged in double rows and last 4 cells arranged in a single row (Fig. 5).

Furthermore, our findings revealed that the gut of *T. axei* 3^rd^ larvae is significantly longer than that of other larvae (411.3 μm ± 53.6), whereas *Cyathostomum s.l.* and *S. westeri* have the shortest gut lengths (225.1 μm ± 41, and 234.1 μm ± 37.2 respectively) (Fig. 2D, Table 1D). However, there was no significant difference in gut length between 3^rd^ larvae of *Cyathostomum s.l.* and *S. westeri* (Table S4).

### Tail sheath

**Fig. 6:**
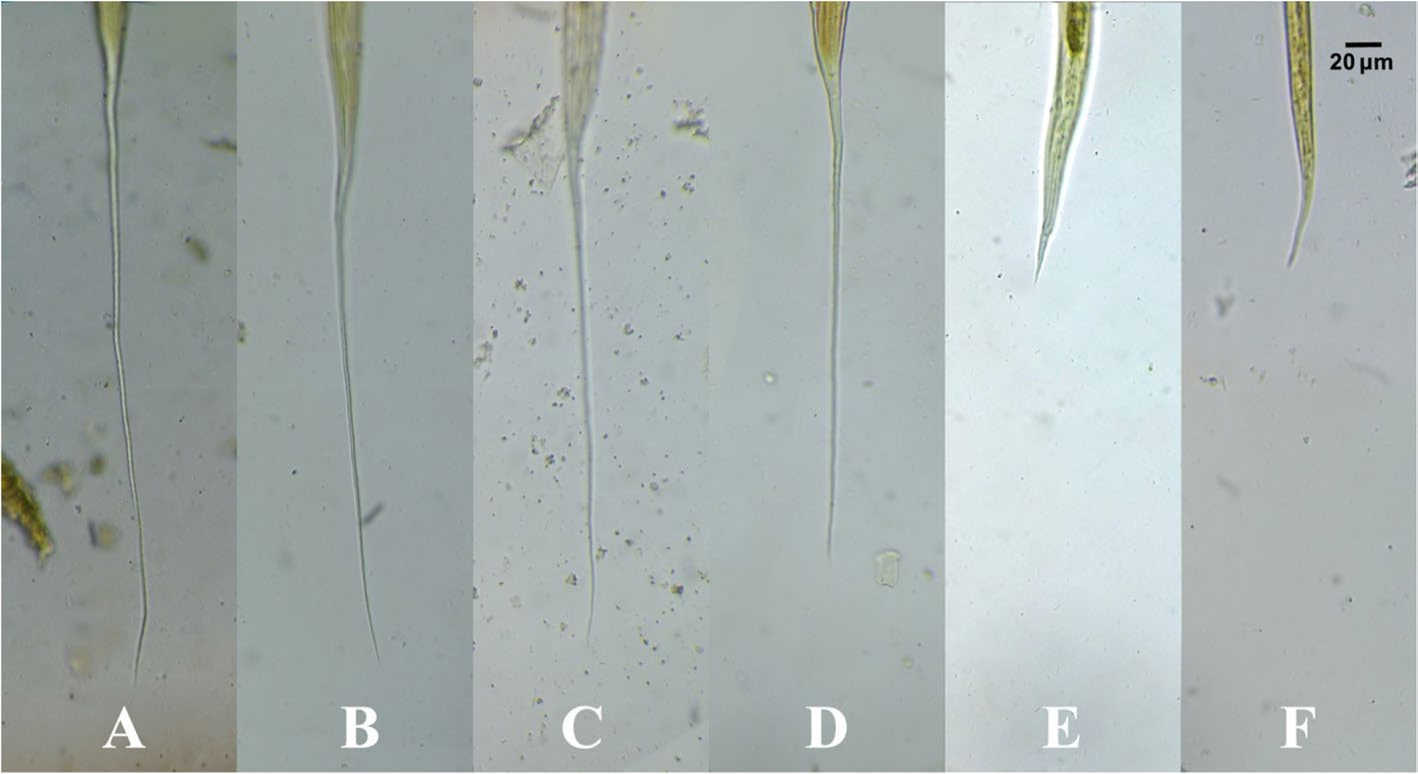
Tail sheath of L3 (Stained with 2% Lugol’s iodine, × 40 magnification, bar = 20 μm). **A** *Cyathostomum* *sensu* *lato*. **B** *Strongylus vulgaris*. **C** *Strongylus equinus*. **D** *Strongylus edentatus*. **E** *Trichostrongylus axei*. **F** *Strongyloides westeri.* All images were taken by the authors

The tail sheath is another feature used to recognize 3^rd^ larvae in this study, with a filamentous tail sheath in *Cyathostomum s.l.*, *S. vulgaris*, *S. equinus*, and *S. edentatus*, a short and non-filamentous tail sheath in *T. axei*, and absence of the tail sheath in *S. westeri* 3^rd^ larvae (Fig. 6).

## Discussion

In the present study, authors noticed that the majority of identified 3^rd^ larvae were originated from donkeys compared to horses (data not shown). Authors interpret this finding based on the hypothesis that donkeys usually receive less attention from their owners and are kept under poor management conditions that enhance the spreading of infection between them. On the other side, horses were less infected since most of them were fed grain byproducts and were less exposed to pasture grazing in addition to the awareness of their owners about the importance of worm control programs.

In the present work, authors identified 3^rd^ larvae of four strongylid nematode species (*Cyathostomum* *sensu* *lato*, *Strongylus vulgaris*, *Strongylus equinus*, *and Strongylus edentatus*), and two non-strongylid nematode species (*Strongyloides westeri*, and *Trichostrongylus axei*). Regarding morphological features, 3^rd^ larvae of *S. vulgaris*, *S. equinus*, *S. edentatus*, *S. westeri*, and *T. axei* were morphologically similar to those described by [14, 15, 18, 19]. Furthermore, morphological features of *Cyathostomum s.l. 3*^*rd*^ larvae were similar to those described by [7, 10].

On the other side, measurement of different dimensions across larvae (A, B1, B2, and C) showed a difference from either those reported in Egypt by [18, 20] or those reported globally by [7, 10, 19]. The reason for the difference in measurements was discussed by [10] who declared that animal breed, nutritional status, and geographic conditions all can contribute to a difference in measurements across different studies. Another factor that could affect measurements was discussed by [21] who reported that culturing temperature either higher or lower than 18–23 °C will lead to a reduction in the length of 3^rd^ larvae. Gugosyan et al. concluded that abiotic factors have a direct effect on the development and morphometric features of embryonic and postembryonic stages of *S. westeri*, and they stated that the optimal temperature for development of *S. westeri* eggs should be 25 °C [22]. Furthermore, according to previous reported study [21], fecal moisture content (FMC) is another factor that has a direct correlation with L3 length, with the more humid culture producing longer L3 and the reason for the variation in length being primarily due to variation in the length of intestinal cells.

In this work, authors noticed that morphological features of intestinal cells in some larvae were superimposed and thus difficult to be counted. Bevilaqua et al. demonstrated the reason for this by revealing that when nematode larvae reach L3, their life span is dependent on food stored in their intestinal cells, and within one-week, intestinal cells become barely distinguishable [19]. As a result, we definitely suggest that morphological identification of intestinal cells of L3 should be followed by measuring their length to confirm the results.

Despite the fact that we were able to identify *S. westeri* infection based on unique morphology of their eggs. The majority of *S. westeri* infections, however, were found in mixed infections with other nematodes (data not shown). As a result, authors intend to culture all positive samples in order to provide extensive data on the morphological features of the most common strongylid and non-strongylid nematodes infecting equines in order to improve their identification in field samples and to evaluate previously existing knowledge about in vitro climatic conditions favorable for the development of these nematodes.

The lack of calculating the length of the posterior end of larvae (from anus to tip of tail sheath) and estimating the ratio of tail length to the rest of the body was a limitation of this study. The study also has limitations in estimating the relative location of anterior intestinal cells in the different types of *Cyathostomum s.l.* that have been identified. We recognized the importance of these points, but they were outside the scope of this study.

In conclusion, even though morphological identification of cultured L3 has some limitations where it is a time-consuming technique (requires 7 days for eggs to give mature L3), liable to bias (since culture temperature and humidity would favor the development of some species of nematode over others), and the identification of cultured L3 based on reference morphological keys requires a lot of training and practice to reach an appropriate level of proficiency. However, regardless of limitations, this technique is considered the cheapest way to identify nematode species infecting livestock. According to the authors’ knowledge, the present work will be among the recent publications describing the morphology of cultured L3 of common strongylid and non-strongylid nematode species infecting equines in Egypt.

## Supplementary Information

**Additional file 1: Table S1.** Multiple Comparisons Test for Total Body Length. **Table S2.** Multiple Comparisons Test for Body Width. **Table S3.** Multiple Comparisons Test for Esophagus Length. **Table S4.** Multiple Comparisons Test for Gut Length.

## Data Availability

The data supporting the findings of this study are contained within the manuscript. The raw data are available from the corresponding author on reasonable request.
