# Supplementary material for: Identifying 3rd larval stages of common strongylid and non-strongylid nematodes (class: Nematoda) infecting Egyptian equines based on morphometric analysis
Source: BMC Vet Res. 2022 Dec 12;18:432. doi: 10.1186/s12917-022-03526-8 (PMC9743504; doi:10.1186/s12917-022-03526-8)
Supplement: Supplementary file 1 — Additional file 1: Table S1. Multiple Comparisons Test for Total Body Length. Table S2. Multiple Comparisons Test for Body Width. Table S3. Multiple Comparisons Test for Esophagus Length. Table S4. Multiple Comparisons Test for Gut Length. [file 12917_2022_3526_MOESM1_ESM.docx]

Table S1 Multiple Comparisons Test for Total Body Length

|  | **95.00% CI of diff.** |  | ***P Value*** | **Significant** |  |
| --- | --- | --- | --- | --- | --- |
| ***Cyathostomum sensu lato*** vs. ***Strongylus vulgaris*** | -97.81 to 22.22 |  | 0.4444 | Ns |  |
| ***Cyathostomum sensu lato*** vs. ***Strongylus equinus*** | -134.8 to -12.23 |  | 0.0097 | ** |  |
| ***Cyathostomum sensu lato*** vs. ***Strongylus edentatus*** | -146.4 to -8.889 |  | 0.0178 | * |  |
| ***Cyathostomum sensu lato*** vs. ***Trichostrongylus axei*** | -69.36 to 80.68 |  | >0.9999 | Ns |  |
| ***Cyathostomum sensu lato*** vs. ***Strongyloides westeri*** | 16.98 to 132.7 |  | 0.0041 | ** |  |
| ***Strongylus vulgaris*** vs. ***Strongylus equinus*** | -100.6 to 29.14 |  | 0.5926 | Ns |  |
| ***Strongylus vulgaris*** vs. ***Strongylus edentatus*** | -111.8 to 32.11 |  | 0.5870 | Ns |  |
| ***Strongylus vulgaris*** vs. ***Trichostrongylus axei*** | -34.51 to 121.4 |  | 0.5801 | Ns |  |
| ***Strongylus vulgaris*** vs. ***Strongyloides westeri*** | 51.01 to 174.3 |  | <0.0001 | **** |  |
| ***Strongylus equinus*** vs. ***Strongylus edentatus*** | -77.15 to 68.91 |  | >0.9999 | Ns |  |
| ***Strongylus equinus*** vs. ***Trichostrongylus axei*** | 0.2321 to 158.1 |  | 0.0489 | * |  |
| ***Strongylus equinus*** vs. ***Strongyloides westeri*** | 85.49 to 211.3 |  | <0.0001 | **** |  |
| ***Strongylus edentatus*** vs. ***Trichostrongylus axei*** | -1.571 to 168.2 |  | 0.0574 | Ns |  |
| ***Strongylus edentatus*** vs. ***Strongyloides westeri*** | 82.32 to 222.7 |  | <0.0001 | **** |  |
| ***Trichostrongylus axei*** vs. ***Strongyloides westeri*** | -7.134 to 145.5 |  | 0.0976 | Ns |  |

Statistical analyses of total body length between different 3^rd^ larvae. Statistically significant differences were determined by a one-way ANOVA and a Tukey-Kramer post hoc analysis (**P* < 0.05). These analyses complement the data shown in Fig. 2A. *CI* Confidence Interval, *Ns* Non-significant

Table S2 Multiple Comparisons Test for Body Width

|  | **95.00% CI of diff.** |  | ***P Value*** | **Significant** |  |
| --- | --- | --- | --- | --- | --- |
| ***Cyathostomum sensu lato*** vs. ***Strongylus vulgaris*** | -2.326 to 4.122 |  | 0.9640 | Ns |  |
| ***Cyathostomum sensu lato*** vs. ***Strongylus equinus*** | -7.737 to -0.8970 |  | 0.0054 | ** |  |
| ***Cyathostomum sensu lato*** vs. ***Strongylus edentatus*** | -8.521 to -0.8311 |  | 0.0083 | ** |  |
| ***Cyathostomum sensu lato*** vs. ***Trichostrongylus axei*** | -2.199 to 6.203 |  | 0.7309 | Ns |  |
| ***Cyathostomum sensu lato*** vs. ***Strongyloides westeri*** | 6.985 to 13.24 |  | <0.0001 | **** |  |
| ***Strongylus vulgaris*** vs. ***Strongylus equinus*** | -8.763 to -1.667 |  | 0.0007 | *** |  |
| ***Strongylus vulgaris*** vs. ***Strongylus edentatus*** | -9.533 to -1.615 |  | 0.0013 | ** |  |
| ***Strongylus vulgaris*** vs. ***Trichostrongylus axei*** | -3.202 to 5.410 |  | 0.9748 | Ns |  |
| ***Strongylus vulgaris*** vs. ***Strongyloides westeri*** | 5.947 to 12.48 |  | <0.0001 | **** |  |
| ***Strongylus equinus*** vs. ***Strongylus edentatus*** | -4.479 to 3.761 |  | 0.9998 | Ns |  |
| ***Strongylus equinus*** vs. ***Trichostrongylus axei*** | 1.865 to 10.77 |  | 0.0012 | ** |  |
| ***Strongylus equinus*** vs. ***Strongyloides westeri*** | 10.97 to 17.89 |  | <0.0001 | **** |  |
| ***Strongylus edentatus*** vs. ***Trichostrongylus axei*** | 1.890 to 11.47 |  | 0.0015 | ** |  |
| ***Strongylus edentatus*** vs. ***Strongyloides westeri*** | 10.91 to 18.67 |  | <0.0001 | **** |  |
| ***Trichostrongylus axei*** vs. ***Strongyloides westeri*** | 3.876 to 12.34 |  | <0.0001 | **** |  |

Statistical analyses of body width between different 3^rd^ larvae. Statistically significant differences were determined by a one-way ANOVA and a Tukey-Kramer post hoc analysis (**P* < 0.05). These analyses complement the data shown in Fig. 2B. *CI* Confidence Interval, *Ns* Non-significant

Table S3 Multiple Comparisons Test for Esophagus Length

|  | **95.00% CI of diff.** |  | ***P Value*** | **Significant** |  |
| --- | --- | --- | --- | --- | --- |
| ***Cyathostomum sensu lato*** vs. ***Strongylus vulgaris*** | 6.453 to 59.75 |  | 0.0065 | ** |  |
| ***Cyathostomum sensu lato*** vs. ***Strongylus equinus*** | -27.07 to 29.46 |  | >0.9999 | Ns |  |
| ***Cyathostomum sensu lato*** vs. ***Strongylus edentatus*** | -30.38 to 33.17 |  | >0.9999 | Ns |  |
| ***Cyathostomum sensu lato*** vs. ***Trichostrongylus axei*** | -34.12 to 35.33 |  | >0.9999 | Ns |  |
| ***Cyathostomum sensu lato*** vs. ***Strongyloides westeri*** | -124.9 to -73.23 |  | <0.0001 | **** |  |
| ***Strongylus vulgaris*** vs. ***Strongylus equinus*** | -61.23 to -2.581 |  | 0.0250 | * |  |
| ***Strongylus vulgaris*** vs. ***Strongylus edentatus*** | -64.43 to 1.018 |  | 0.0630 | Ns |  |
| ***Strongylus vulgaris*** vs. ***Trichostrongylus axei*** | -68.08 to 3.098 |  | 0.0936 | Ns |  |
| ***Strongylus vulgaris*** vs. ***Strongyloides westeri*** | -159.2 to -105.2 |  | <0.0001 | **** |  |
| ***Strongylus equinus*** vs. ***Strongylus edentatus*** | -33.86 to 34.26 |  | >0.9999 | Ns |  |
| ***Strongylus equinus*** vs. ***Trichostrongylus axei*** | -37.40 to 36.23 |  | >0.9999 | Ns |  |
| ***Strongylus equinus*** vs. ***Strongyloides westeri*** | -128.8 to -71.68 |  | <0.0001 | **** |  |
| ***Strongylus edentatus*** vs. ***Trichostrongylus axei*** | -40.36 to 38.79 |  | >0.9999 | Ns |  |
| ***Strongylus edentatus*** vs. ***Strongyloides westeri*** | -132.5 to -68.40 |  | <0.0001 | **** |  |
| ***Trichostrongylus axei*** vs. ***Strongyloides westeri*** | -134.7 to -64.69 |  | <0.0001 | **** |  |

Statistical analyses of esophagus length between different 3^rd^ larvae. Statistically significant differences were determined by a one-way ANOVA and a Tukey-Kramer post hoc analysis (**P* < 0.05). These analyses complement the data shown in Fig. 2C. *CI* Confidence Interval, *Ns* Non-significant

Table S4 Multiple Comparisons Test for Gut Length

|  | **95.00% CI of diff.** |  | ***P Value*** | **Significant** |  |
| --- | --- | --- | --- | --- | --- |
| ***Cyathostomum sensu lato*** vs. ***Strongylus vulgaris*** | -150.4 to -66.75 |  | <0.0001 | **** |  |
| ***Cyathostomum sensu lato*** vs. ***Strongylus equinus*** | -98.57 to -9.789 |  | 0.0080 | ** |  |
| ***Cyathostomum sensu lato*** vs. ***Strongylus edentatus*** | -112.1 to -12.32 |  | 0.0063 | ** |  |
| ***Cyathostomum sensu lato*** vs. ***Trichostrongylus axei*** | -240.8 to -131.8 |  | <0.0001 | **** |  |
| ***Cyathostomum sensu lato*** vs. ***Strongyloides westeri*** | -50.12 to 32.22 |  | 0.9879 | Ns |  |
| ***Strongylus vulgaris*** vs. ***Strongylus equinus*** | 8.370 to 100.5 |  | 0.0113 | * |  |
| ***Strongylus vulgaris*** vs. ***Strongylus edentatus*** | -5.008 to 97.76 |  | 0.1004 | Ns |  |
| ***Strongylus vulgaris*** vs. ***Trichostrongylus axei*** | -133.6 to -21.83 |  | 0.0016 | ** |  |
| ***Strongylus vulgaris*** vs. ***Strongyloides westeri*** | 56.69 to 142.6 |  | <0.0001 | **** |  |
| ***Strongylus equinus*** vs. ***Strongylus edentatus*** | -61.52 to 45.44 |  | 0.9978 | Ns |  |
| ***Strongylus equinus*** vs. ***Trichostrongylus axei*** | -190.0 to -74.32 |  | <0.0001 | **** |  |
| ***Strongylus equinus*** vs. ***Strongyloides westeri*** | -0.2099 to 90.66 |  | 0.0518 | Ns |  |
| ***Strongylus edentatus*** vs. ***Trichostrongylus axei*** | -186.2 to -61.95 |  | <0.0001 | **** |  |
| ***Strongylus edentatus*** vs. ***Strongyloides westeri*** | 2.430 to 104.1 |  | 0.0346 | * |  |
| ***Trichostrongylus axei*** vs. ***Strongyloides westeri*** | 122.0 to 232.7 |  | <0.0001 | **** |  |

Statistical analyses of gut length between different 3^rd^ larvae. Statistically significant differences were determined by a one-way ANOVA and a Tukey-Kramer post hoc analysis (**P* < 0.05). These analyses complement the data shown in Fig. 2D. *CI* Confidence Interval, *Ns* Non-significant
